# The complete mitochondrial genome of the cavity-nesting honeybee, *Apis cerana* (Insecta: Hymenoptera: Apidae) from Borneo

**DOI:** 10.1080/23802359.2017.1361344

**Published:** 2017-07-31

**Authors:** Hisashi Okuyama, Salim Tingek, Jun-ichi Takahashi

**Affiliations:** a Department of Life sciences, Kyoto Sangyo University, Kyoto, Japan;; b Agriculture Research Station Tenom, Tenom, Malaysia

**Keywords:** Asian honeybee, Illumina sequencing, genetic distance, *Apis cerana*, Borneo

## Abstract

The complete mitochondrial genome of the cavity-nesting honeybee *Apis cerana* from Sabah on Borneo Island was analyzed using next-generation sequencing. The mitochondrial genome of *A. cerana* was a circular molecule of 15,884 bp and was similar to that of the other cavity-nesting honeybee species. The average AT content in the *A. cerana* mitochondrial genome was 84.4%. It was predicted to contain 13 protein-coding, 22 tRNA, and two rRNA genes, along with one A + T-rich control region.

The Asian cavity-nesting honeybee, *Apis cerana,* is widely distributed in the Asian continent and surrounding islands. Multivariate morphometric analysis of *A. cerana* suggested the presence of four or more subspecies (Ruttner 1988). Mitochondrial DNA analyses of the partial DNA sequences indicated that *A. cerana* from Borneo has higher genetic diversity compared to that in Asian continent populations (Smith and Hagen 1996; Smith et al. 2000; Takahashi et al. 2002; Tanaka et al. 2003; Arias and Sheppard 2005), but its phylogenetic position remains uncertain (Tanaka et al. 2001; Lo et al. 2010). To our knowledge, this is the first study to analyze the complete mitochondrial genome of *A. cerana* from Borneo.

Adult workers of *A. cerana* in Sabah, Malaysia, were collected in March 2000 (the specimen was stored in the National Museum of Nature and Science, Japan, accession number: NSMT-I-HYM74241). Genomic DNA isolated from worker was sequenced using Illumina’s HiSeq platform. The resultant reads were assembled and annotated using the MITOS web server (Germany; Bernt et al. 2013) and Geneious R9 (Biomatters, Auckland, New Zealand). A phylogenetic tree was constructed using MEGA6 (Tamura et al. 2013) and TREEFINDER v.2011 (Jobb et al. 2004) by using the nucleotide sequences of the 13 protein-coding genes.

The *A. cerana* mitochondrial genome was found to form a closed loop that is 15,884 bp long (AP018149). The *A. cerana* mitochondrial genome represented a typical hymenopteran pattern and was similar to the common *A. cerana* mitochondrial genome organization, comprising 13 protein-coding genes, 22 putative tRNA genes, two rRNA genes, and an A + T-rich control region. The average AT content of the *A. cerana japonica* mitochondrial genome was 84.36%. Similar to honeybee mitochondrial genomes, the heavy strand encoded nine protein-coding genes and 14 tRNA genes, and the light strand encoded four protein-coding genes, eight tRNAs, and two rRNA genes. The *ATP6* and *ATP8* genes shared 19 nucleotides. Nine protein-coding genes of the *A. cerena* mitochondrial genome started with ATT; *ATP6*, *COIII*, and *Cytb* genes, with ATG; and *ATP8* gene, with ATC, all of which have been commonly found in the *A. cerana* mitochondrial genome (Tan et al. 2011; Takahashi et al. 2016; Okuyama et al. 2017). The stop codon of each of these protein-coding genes was either TAA, similar to the case in other honeybees. All of the tRNA genes typically possessed cloverleaf secondary structures, except for *tRNA-Ser*, which lacked the dihydrouridine arm.

Phylogenetic analysis was conducted using 13 mitochondrial protein-coding genes with 19 closely related taxa (Figure 1). The genetic distance and mutation site of 13 mitochondrial protein-coding genes in the *A. cerana* subspecies mitochondrial genome were 0.0195 and 206 on average, respectively. The Bornean *A. cerana* was closer to *A. cerana cerana* than to *A. cerana japonica*, consistent with the findings of previous multivariate morphometric studies (Radloff et al. 2010). The complete mitochondrial DNA sequence of *A. cerana* has only been identified for seven populations, including that from Borneo. However, the complete mitochondrial DNA sequence provides additional valid information for assessing the phylogeny of this group.

**Figure 1.**
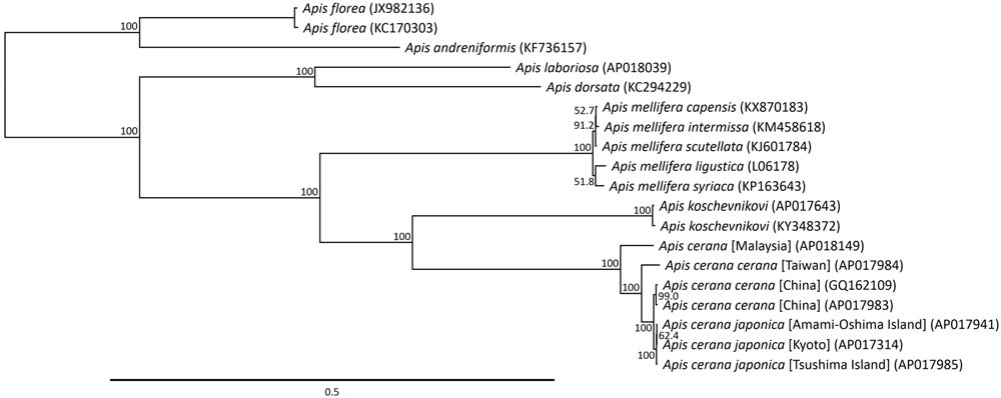
Phylogenetic relationships (maximum likelihood) among the species of the genus *Apis* (Hymenoptera) determined using the mitochondrial DNA nucleotide sequences of the 13 protein-coding genes. Numbers beside the nodes are percentages of 1000 bootstrap values. *Apis florea* and *Apis andreniformis* were used as an out-group. Alphanumeric terms in parentheses indicate the GenBank accession numbers.
